# Temporal trends of suicide-related non-traumatic out-of-hospital cardiac arrest characteristics and outcomes with the COVID-19 pandemic

**DOI:** 10.1016/j.resplu.2022.100216

**Published:** 2022-03-03

**Authors:** Justin Yap, Frank X. Scheuermeyer, Sean van Diepen, David Barbic, Ron Straight, Nechelle Wall, Michael Asamoah-Boaheng, Jim Christenson, Brian Grunau

**Affiliations:** a British Columbia Resuscitation Research Collaborative, British Columbia, Canada; b Faculty of Science, University of British Columbia, British Columbia, Canada; c Department of Emergency Medicine, University of British Columbia, British Columbia, Canada; d Centre for Health Evaluation and Outcome Sciences, British Columbia, Canada; e Division of Critical Care, University of Alberta, Alberta, Canada; f British Columbia Emergency Health Services, British Columbia, Canada

## Abstract

**Background:**

Jurisdictions have reported COVID-19-related increases in the incidence and mortality of non-traumatic out-of-hospital cardiac arrest (OHCA). We hypothesized that changes in suicide incidence during the COVID-19 pandemic may have contributed to these changes. We investigated whether the COVID-19 pandemic was associated with changes in the: (1) incidence of suicide-related OHCA, and (2) characteristics and outcomes of such cases.

**Methods:**

We used the provincial British Columbia Cardiac Arrest Registry, including non-traumatic emergency medical system (EMS)-assessed OHCA, to compare suicide-related OHCA (defined as clear self-harm or *a priori* communication of intent) one-year prior to, and one year after, the start of the COVID-19 pandemic (March 15, 2020). We calculated differences in incidence (with 95% CI), overall and within subgroups of mechanism (hanging, suffocation, poisoning, or unclear mechanism), and in case characteristics and hospital-discharge favourable neurological outcomes (CPC 1–2).

**Results:**

Of 13,785 EMS-assessed OHCA, we included 274/6430 (4.3%) pre-pandemic and 221/7355 (3.0%) pandemic-period suicide-related cases. The median age was 43 years (IQR 30–57), 157 (32%) were female, and 7 (1.4%) survived with favourable neurological status. Suicide-related OHCA incidence decreased from 5.4 pre-pandemic to 4.3 per 100 000 person-years (-1.1, 95% CI −2.0 to −0.28). Hanging-related OHCA incidence also decreased. Patient characteristics and hospital discharge outcomes between periods were similar.

**Conclusion:**

Suicide-related OHCA incidence decreased with the COVID-19 pandemic and we did not detect changes in patient characteristics or outcomes, suggesting that suicide is not a contributor to increases in COVID-related OHCA incidence or mortality. Overall suicide-related OHCA outcomes in both time periods were poor.

## Introduction

Emergency medical service (EMS) personnel in North America assess over 350,000 individuals with non-traumatic out-of-hospital cardiac arrest (OHCA) annually.^1^ While the predominant etiology of OHCA are cardiac events,2., 3. registries have reported that non-cardiac etiology-OHCAs have been trending upwards in recent years.4., 5., 6. Within this subset include those with hanging, asphyxiation, and poisoning, of which survival and neurological outcomes are relatively poor7., 8. compared to cardiac-related events.^9^ Suicide, which may account for a proportion of these cases, is a multifaceted process involving interactions between neurobiology, times of crisis, social and cultural factors.^10^ Suicide is the second leading cause of death among young individuals, responsible for 700 000 deaths per year globally.^11^

Several communities have reported that the COVID-19 pandemic has increased the incidence and mortality of non-traumatic OHCA.^12^ It is possible that changes in the incidence of suicide may have, in part, played a role in these changes. The COVID-19 pandemic has worsened community and individual isolation, fear, uncertainty, psychological disorders, economic hardship, and marginalization, and such conditions may increase the risk of suicide.^13^ While previous studies have described the negative impact on the mental health due to the COVID-19 pandemic,14., 15., 16., 17. investigations looking at the early phase of the pandemic have not seen increases in suicide.^18^ However, the incidence may change as the pandemic continues for longer periods. Further, characteristics of individuals with suicide-related OHCAs may been affected by the pandemic—Japan reported a shift in suicides toward younger individuals and females in the second wave.^19^.

Given the potentially changing epidemiology of suicide due COVID-19, as well as the grim survival statistics, we investigated whether the clinical epidemiology (demographics and mechanism) and outcomes of EMS-assessed suicide-related non-traumatic OHCA changed during the COVID-19 pandemic, in comparison to prior to the pandemic. We sought to investigate whether suicide may be playing a role in observed increases in OHCA incidence and mortality in registry data.^12^ We hypothesized that suicide incidence increased during the COVID-19 pandemic and that outcomes of suicide-related OHCAs worsened due to social isolation.

## Methods

### Study setting & design

This observational study used data from the British Columbia (BC) Cardiac Arrest Registry from March 15, 2019 to March 14, 2021 The registry prospectively identifies consecutive non-traumatic OHCAs assessed by the single provider of out-of-hospital medical care in BC (BC Emergency Health Services [BCEHS]), and abstracts data including bystander interventions, time-stamped EMS-interventions, etiology, and outcomes (based on recommended Utstein variables^20^) into a RedCap Database.21., 22. The registry does not include cases of major trauma (defined as due to blunt, penetrating, or burn-related injuries), however all other etiologies of OHCA are included (including hanging, asphyxiation, smoke inhalation, etc.). BC had a population of 5,046,576 in 2019 and 5,153,039 in 2021.^23^

## EMS medical care

EMS response to OHCAs in BC is initiated by a 9-1-1 call to a dispatcher, who initiates a co-ordinated response from municipal fire departments (FD) and BCEHS. In the event of an OHCA, the following EMS units typically get dispatch to the scene: FD, a primary care paramedic (PCP) BCEHS unit, and an advance care paramedic (ACP) BCEHS unit. Dispatchers instruct bystanders on scene to perform cardiopulmonary resuscitation (CPR) and apply an automated external defibrillator (AED). Upon of EMS units, personnel perform or withhold resuscitation based upon BCEHS policies (Appendix A, with COVID-19-related updates in Appendix B).

## Selection of participants and comparison periods

We included suicide-related (definition below) OHCAs (both EMS-treated and untreated) from the BC Cardiac Arrest Registry. We dichotomized cases into pre-COVID-19 pandemic (March 15, 2019 to March 14, 2020) or during the pandemic (March 15, 2020 to March 15, 2021) based on event date. The COVID-19 pandemic timeline was set to start on March 15, 2020, the date the BC government closed non-essential services and limiting physical gatherings.

## Data collection and variable definitions

From March 15, 2019 and March 15, 2021, we added a “suicide-related OHCA” binary variable to the BC Cardiac Arrest Registry. Using information recorded on the prehospital clinical record, registry staff identified suicide-relate cases, defined as: (1) the patient communicated intent for self-harm, either to others (up to seven days prior to the OHCA) or in written form; or, (2) evidence of self-harm, including cases that paramedics found patient hanging, a bag over the head or an object wrapped tightly around the neck, ingestion of toxic chemicals (e.g., bleach), or inhalation of toxic gases in a small sealed environment (e.g. vehicle exhaust redirected into a locked vehicle). The BC Cardiac Arrest Registry has routinely classified and recorded the etiology of obvious non-cardiac OHCAs.^24^ We used these categories to further describe suicide-related cases, grouping into four categories: hanging, suffocation, poisoning, or unclear. Hangings were defined as patients found by EMS or bystanders suspended above the ground with an object wrapped around their neck. Suffocation included non-hanging mechanical asphyxiation (e.g., bag securely positioned over head or item wrapped around neck) and drowning. Poisoning included chemicals (e.g., industrial gases, cleaning solutions, vehicle exhaust fumes) or drug (illicit or prescription) via any route of administration. Other cases were classified as “unclear”.

For all records, we examined age, sex, EMS treatment status (treated or untreated) and time of the incident (0600 – 1159, 1200 – 1759, 1800-2359, and 0000 – 0559). For EMS-treated cases we reported characteristics including EMS response time (9-1-1 call-to-EMS on-scene arrival^25^), first arriving EMS unit (FD, primary care paramedic [PCP], advance care paramedic [ACP]), bystander CPR, witness status (bystander witnessed, EMS witnessed, unwitnessed), return of spontaneous circulation (ROSC), initial cardiac rhythm (non-shockable [asystole, pulseless electrical activity [PEA], unspecified non-shockable], shockable [ventricular fibrillation [VF] or pulseless ventricular tachycardia], no automated external defibrillator (AED) applied), final status with EMS (termination of resuscitation on scene, or transported to the hospital), and hospital-discharge outcomes.^26^ For untreated OHCA, we reported reasons for withholding resuscitation, including: DNR (do not resuscitate) order, terminal illness, verbal directive (family of the patient verbally requested no resuscitation), obvious death (where the patient shows signs of rigor mortis, lividity, tissue decomposition or injuries incompatible with life), prolonged period from the cardiac arrest until EMS arrival without signs of obvious death, or unspecified (no reason noted in the paramedic report).

## Outcomes

The primary outcome was suicide-related OHCA. Secondary outcomes included survival and with favourable neurological outcome (defined as cerebral performance category 1–2) at hospital discharge.^20^

### Data analysis

We captured data using RedCap (Vanderbilt, Nashville).^22^ We performed data analysis using Microsoft Excel 16.42 (Microsoft Corp, Redmond), MedCalc Statistical Software version 19.2.6 (MedCalc Software, Ostend, Belgium), and R (Foundation for Statistical Computing, Vienna, version 3.2.4). We expressed categorical variables as proportions with confidence intervals and continuous variables as means with standard deviations. Using the total BC population, we calculated the incidence rate per 100 000 person-years prior to, and during, the COVID-19 pandemic. We compared the difference of suicide-related OHCA (with a 95% confidence interval [CI]) overall, and within the categories based on sex, mechanism of suicide, and EMS-treatment. We also compared between-time period case characteristics, using the total suicide-related OHCA cohort as the denominator, and stratified by mechanism of suicide. Continuous variables compared using a t-test.

## Results

Between March 15, 2019 and March 15, 2021, we identified 13,785 EMS-assessed OHCAs, of which 495 (3.6%) were suicide-related. Overall, 157 (32%) were female, the median age was 43 (IQR 30 to 57), 399 (81%) cases involved hanging, and 224 (45%) were EMS-treated. Of the EMS-treated cases, 215 (96%) had an initial non-shockable cardiac rhythm, 55 (25%) were transported to the hospital, 8 (3.6%) survived to hospital discharge, with 7 (3.1%) having favourable neurological outcomes. Of the 271 non-treated patients, 263 (97%) exhibited signs of obvious death.

Population-level suicide-related OHCA incidence rates, overall and categorized by sex, mechanism, and EMS-treatment status are shown in Table 1. Overall suicide-related OHCA incidence decreased from 5.4 per 100,000 pre-pandemic, to 4.3 per 100,000 during the pandemic (difference −1.1 per 100 000 persons-years, 95% CI −2.0, −0.28). Hanging-related cases (difference −0.96 per 100 000 persons-years, 95% CI −1.7, −0.20) and cases of males (difference −0.72 per 100 000 persons-years, 95% −1.4, −0.0012) also decreased.

**Table 1:** Suicide related OHCA incidence Pre-COVID versus COVID time – total suicides, patient demographics, suicide mechanism, EMS treated/untreated.

		**Suicide related OHCA Incidence**							

	Pre-Pandemic Period								
(population = 5,046,576)	Incidence rate pre-Pandemic per 100 000 annually	Missing	Pandemic Period						
(n = 5153039)	Incidence rate Pandemic per 100 000 annually	Missing							
	Difference (with 95% CI) per								
100 000 annually									
**Total Suicides**	274	5.4	–	221	4.3	–	−1.1 (−2.0, −0.28)		
**Demographics**									
Male	184	3.6	3	151	2.9	–	−0.72 (−1.4, −0.0012)		
Female	87	1.7		70	1.4		−0.37 (−0.85, 0.12)		
**Mechanism**			–			–			
Hanging	222	4.4		177	3.4		−0.96 (−1.7, −0.20)		
Poisoning	26	0.52		27	0.52		0.0088 (−0.27, 0.29)		
Suffocation	22	0.44		13	0.25		−0.18 (−0.41, 0.044)		
Unclear	4	0.079		4	0.078		−0.0016 (−0.11, 0.11)		
**Treated by EMS**	106	2.1	–	118	2.3	–	0.19 (−0.39, 0.76)		
**Untreated by EMS**	168	3.3	–	103	2.0	–	−1.3 (−2.0, −0.70)		

OHCA, out of hospital cardiac arrest; Pre-Pandemic, time period before COVID-19 in British Columbia (March 15, 2019 to March 14, 2020); Pandemic, time period during COVID-19 in British Columbia (March 15, 2020 to March 15 2021); CI, confidence interval; Poisoning, chemical or drug ingestion/inhalation/injection; Suffocation, asphyxiation/drowning; EMS, Emergency Medical Services.

The proportion of EMS-assessed OHCAs that were suicide-related decreased from 274/6430 (4.3%) pre-pandemic, to 221/7355 (3.0%) during the pandemic (difference −1.3, 95% CI −1.9, −0.63). Table 2 compares the characteristics among suicide-related OHCAs stratified by time period. Comparing the pre-pandemic to pandemic periods, there was an increase in the proportion of EMS-treated cases (15%, 95% CI 6.0 to 23), however other characteristics and outcomes were similar.

**Table 2:** Total suicide related OHCA Pre COVID versus COVID time – patient demographics, mechanism of suicide, incident characteristics, patient outcomes (survival rate and CPC score).

**Total Suicide related OHCA**					
	Pre-Pandemic Period (n = 274)	Missing (%)	Pandemic Period (n = 221)	Missing (%)	Risk Difference (95% CI)
Mean Age (SD), y	46 (20)	10 (3.6)	43 (18)	6 (2.7)	−2.7 (−6.1, 0.63)
Female (%)	87 (32)		70 (32)		−0.43 (−8.7, 7.8)

Time		0 (0)		0 (0)	
Morning (%)	78 (28)		72 (32)		3.7 (−4.5, 12)
Afternoon (%)	107 (39)		74 (33)		−5.6 (−14, 2.9)
Evening (%)	70 (26)		62 (28)		2.5 (−5.4, 10)
Night (%)	19 (6.9)		14 (6.3)		−0.60 (−5.0, 3.8)

Mechanism		0 (0)		0 (0)	
Hanging (%)	222 (81)		177 (80)		−0.93 (−8.0, 6.1)
Poisoning (%)	26 (9.5)		27 (12)		2.7 (−2.8, 8.3)
Suffocation & Drowning (%)	22 (8.0)		13 (5.9)		−2.1 (−6.6, 2.3)
Unclear (%)	4 (1.5)		4 (1.8)		0.35 (−1.9, 2.6)

**Untreated by EMS**	168 (61)	−	103 (47)	−	−15 (–23, −6.0)
Reasons for non-treatment		0 (0)		0 (0)	
DNR	3 (1.8)		0 (0)		−1.8 (−3.8, 0.22)
Terminal illness	0 (0)		0 (0)		–
Verbal directive	0 (0)		0 (0)		–
Obvious death	161 (96)		102 (99)		3.2 (−0.37, 6.8)
Prolonged down time	3 (1.8)		1 (0.97)		−0.81(−3.6, 1.9)
Unspecified	1 (0.60)		0 (0)		−0.60 (−1.8, 0.57)

**Treated by EMS**	106 (39)	–	118 (53)	–	15 (6.0, 23)

Mean EMS response time (SD)	10 (9.8)	0 (0)	9.8 (6.4)	0 (0)	−0.51 (−2.7, 1.6)

First on Scene		0 (0)		0 (0)	
Fire (%)	42 (40)		62 (53)		13 (−0.037, 26)
BLS (%)	51 (48)		52 (44)		−4.0 (−17, 9.0)
ALS (%)	13 (12)		4 (3.4)		−8.9 (−16, −1.8)
Witnessed Status		0 (0)		0 (0)	
Bystander Witnessed	3 (2.8)		1 (0.85)		−2.0 (−5.5, 1.6)
Unwitnessed	103 (97)		117 (99)		2.0 (−1.6, 5.5)
EMS/Fire witnessed	0 (0)		0 (0)		–

		3 (2.8)		1 (0.85)	
Bystander CPR	69 (67)		82 (70)		3.1 (−9.2, 15)
		0 (0)		0 (0)	
ROSC	17 (16)		30 (25)		9.4 (−1.1, 20)

Initial Cardiac Rhythm		3 (2.8)		0 (0)	
Non shockable	100 (97)		115 (97)		0.37 (−3.9, 4.7)
Shockable	1 (0.97)		1 (0.85)		−0.12 (−2.6, 2.4)
No AED	2 (1.9)		2 (1.7)		−0.25 (−3.8, 3.3)

Final status with EMS		0 (0)		0 (0)	
Pronounced dead/TOR at scene	83 (78)		86 (72)		−5.4 (−17, 5.8)
Transported to hospital	23 (22)		32 (27)		5.4 (−5.8, 17)

Survival at hospital discharge	6 (5.7)	0 (0)	2 (1.7)	0 (0)	−4.0 (−8.9, 1.0)

CPC score 1–2 at hospital discharge	5 (4.7)	0 (0)	2 (1.7)	0 (0)	−3.0 (−7.7, 1.6)

OHCA, out of hospital cardiac arrest; EMS, Emergency medical service; Pre-Pandemic, time period before COVID-19 in British Columbia (March 15, 2019 to March 14, 2020); Pandemic, time period during COVID-19 in British Columbia (March 15, 2020 to March 15 2021); CI, confidence interval; Poisoning, chemical or drug ingestion/inhalation/injection; Suffocation, asphyxiation/drowning; SD, standard deviation; y, years; Morning, 6:00:00AM-11:59:59 AM; Afternoon, 12:00:00 PM-17:59:59; Evening, 18:00:00–23:59:59; Night, 12:00:00AM-5:59:59AM; EMS response time, interval of time from the 9–1-1 call received by dispatch to EMS scene arrival; Fire, fire department; BLS, Basic life support paramedic; ALS, Advanced life support paramedic; DNR, do-not-resuscitate order; Verbal directive, Family requested no resuscitation verbally; CPC, cerebral performance category; CPR, cardiopulmonary resuscitation; ROSC, return of spontaneous circulation; Non-shockable rhythms, pulseless electrical activity (PEA), asystole; Shockable rhythms, ventricular fibrillation (VF), ventricular tachycardia (VT); TOR, termination of resuscitation; AED, automated external defibrillator; min, minutes.

Case characteristics grouped by mechanism and stratified by time period can be seen in Table 3. Among cases with hangings, the proportion treated by EMS increased in the pandemic era. The poisoning subgroup had the greatest proportion of patients surviving to hospital discharge pre-pandemic (n = 3, 33%) and during COVID-19 (n = 1, 11%). Within the unclear mechanism group there was an increase of cases in females. While suicide-related OHCA typically occurred in the afternoon, suffocation and poisoning subgroups experienced an increase in the evening and night events, respectively. Otherwise, characteristics were similar between groups.

**Table 3:** Suicide-related OHCA Characteristics, EMS Treatment, and Outcomes, Stratified by Mechanism and Pre-Pandemic versus Pandemic Time Period.

**Mechanism of suicide related OHCA**												
	**Hanging (n = 399)**			**Suffocation & Drowning (n = 35**)			**Poisoning (n = 53)**			**Unclear (n = 8)**		
	Pre-Pandemic Period (n = 222)	Pandemic Period (n = 177)	Risk Difference (95% CI)	Pre-Pandemic Period (n = 22)	Pandemic Period (n = 13)	Risk Difference (95% CI)	Pre-Pandemic Period (n = 26)	Pandemic Period (n = 27)	Risk Difference (95% CI)	Pre-Pandemic Period (n = 4)	Pandemic Period (n = 4)	Risk Difference (95% CI)
**Demographics**												
Mean age (SD), y	44 (19)	42 (18)	−1.8 (−5.5, 1.8)	59 (23)	44 (19)	−15 (−31, 0.32)	51 (22)	51 (15)	0.48 (−10, 11)	58 (7.1)	35 (15)	–23 (−43, 2.7)
Male (%)	156 (71)	122 (69)	−2.0 (−11, 7.1)	14 (64)	10 (77)	13 (−17, 44)	10 (40)	17 (63)	23 (−3.5, 49)	4 (100)	2 (50)	−50 (−99, −1.0)
Female (%)	64 (29)	55 (31)	2.0 (−7.1, 11)	8 (36)	3 (23)	−13 (−44, 17)	15 (60)	10 (37)	–23 (−49, 3.5)	0 (0)	2 (50)	50 (1.0, 99)

**Time**												
Morning (%)	62 (28)	61 (34)	6.5 (−2.6, 16)	7 (32)	2 (15)	−16 (−44, 11)	8 (31)	7 (26)	−4.8 (−29, 19)	1 (25)	1 (25)	0 (−60, 60)
Afternoon (%)	85 (38)	58 (33)	−5.5 (−15, 3.9)	10 (45)	5 (38)	−7.0 (−41, 27)	11 (42)	9 (33)	−9.0 (−35, 17)	1 (25)	2 (50)	25 (−40, 90)
Evening (%)	59 (27)	48 (27)	0.54 (−8.2, 9.3)	3 (14)	6 (46)	33 (1.9, 63)	7 (27)	7 (26)	−1.0 (−25, 23)	1 (25)	1 (25)	0 (−60, 60)
Night (%)	16 (7.2)	10 (5.6)	−1.6 (−6.4, 3.3)	2 (9.1)	0 (0)	−9.1 (−21, 2.9)	0 (0)	4 (15)	15 (1.4, 28)	1 (25)	1 (25)	0 (−60, 60)

**Untreated by EMS**	134 (60)	76 (43)	−17 (−27, −7.7)	13 (59)	5 (38)	−21 (−54, 13)	17 (65)	18 (67)	1.3 (−24, 27)	4 (100)	4 (100)	−
Reason for no treatment												
DNR	0 (0)	0 (0)	–	1 (7.7)	0 (0)	−7.7 (–22, 6.8)	1 (5.9)	0 (0)	−5.9 (−17, 5.3)	1 (25)	0 (0)	−25 (−67, 17)
Terminal illness	0 (0)	0 (0)	–	0 (0)	0 (0)	–	0 (0)	0 (0)	–	0 (0)	0 (0)	–
Verbal directive	0 (0)	0 (0)	–	0 (0)	0 (0)	–	0 (0)	0 (0)	–	0 (0)	0 (0)	–
Obvious death	131 (98)	75 (99)	0.92 (−2.7, 4.5)	12 (92)	5 (100)	7.7 (−6.8, 22)	16 (94)	18 (100)	5.9 (−5.3, 17)	2 (50)	4 (100)	50 (1.0, 99)
Prolonged down time	3 (2.2)	1 (1.3)	−0.92 (−4.5, 2.7)	0 (0)	0 (0)	–	0 (0)	0 (0)	–	0 (0)	0 (0)	–
Unspecified	0 (0)	0 (0)	–	0 (0)	0 (0)	–	0 (0)	0 (0)	–	1 (25)	0 (0)	−25 (−67, 17)

**Treated by EMS**	88 (40)	101 (57)	17 (7.7, 27)	9 (41)	8 (62)	21 (−13, 54)	9 (35)	9 (33)	−1.3 (−27, 24)	0 (0)	0 (0)	–

Hospital Discharge Survival	2 (2.3)	1 (1.0)	−1.3 (−4.9, 2.4)	1 (11)	0 (0)	−11 (–32, 9.4)	3 (33)	1 (11)	–22 (−59, 15)	0 (0)	0 (0)	–

CPC score 1–2	1 (1.1)	1 (1.0)	−0.15 (−3.1, 2.8)	1 (11)	0 (0)	−11 (–32, 9.4)	3 (33)	1 (11)	–22 (−59, 15)	0 (0)	0 (0)	–

OHCA, out of hospital cardiac arrest; EMS, Emergency medical service; Pre-Pandemic Period, time period before COVID-19 in British Columbia (March 15, 2019 to March 14, 2020); Pandemic Period, time period during COVID-19 pandemic in British Columbia (March 15, 2020 to March 15 2021); Mis, missing; Poisoning, chemical or drug ingestion/inhalation/injection; SD, standard deviation; y, years; Morning, 6:00:00AM-11:59:59 AM; Afternoon, 12:00:00 PM-17:59:59; Evening, 18:00:00–23:59:59; Night, 12:00:00AM-5:59:59AM; DNR, do-not-resuscitate order; Verbal directive, Family requested no resuscitation verbally; CPC, cerebral performance category; min, minutes.

## Discussion

We reviewed over 13,000 cardiac arrests from a provincial registry of non-traumatic OHCA and identified nearly 500 OHCAs with a suicide-related etiology in a two-year period. While suicide-related OHCA accounts for a low proportion of overall OHCA, patients are young and outcomes are universally poor. We compared suicide-related OHCAs between the pre-COVID-19 and COVID-19 pandemic time periods. Contrary to our hypothesis, we found a decrease of one suicide per 100 000 person-years, and did not see a statistically significant worsening of outcomes.

Our findings showing a decline in suicide-related OHCA incidence are aligned with another study that reported the suicide rate during the 1st wave of the COVID-19 pandemic (March to August 2020).^27^ Another study analyzed suicide trends in 21 countries during the early phase of the COVID-19, and reported the majority of countries experienced no change or a decline in suicide rates, with the exception of Japan, Austria, and Puerto Rico.^18^ Japan’s suicide trend in the first 5 months (February to June 2020) of the pandemic showed a decrease in suicide rate, while data from the following 4 months (July to October 2020) displayed an increase, especially in younger individuals and females.^19^ This differs from our data, which showed no significant changes in females, and a decreased rate of suicide for males. Our findings are consistent with prior work, demonstrating rates of suicide in males to be approximately twice that of females.28., 29..

While our cohort examined suicide-related OHCAs, trends can be compared to studies that have reported changes of overall OHCAs with the COVID-19 pandemic. In our study of suicide-related cases, the proportion of EMS-treated cases increased during COVID-19. This contrasts to a meta-analysis showing no difference in overall EMS-treatment rates during the pandemic,^12^ and another systematic review reporting a decrease in resuscitation attempts during the pandemic.^30^ Our data does not show reduced EMS response times or changes in bystander CPR, which may influence a paramedic’s decision of whether to start resuscitation. Studies examining OHCAs during the earlier phase of the COVID-19 pandemic reported heterogenous results in terms of changes in bystander CPR, ROSC, incidence, survival rate, witness status and EMS response time.12., 30., 31., 32., 33. These differences in findings may be attributed to differences in COVID-19 incidence, community demographics, governmental policies, and EMS protocol changes. Of closest resemblance to our study date range, a study done in Bologna, Italy from January 2020 to June 2020 found no significant difference in bystander CPR, ROSC, EMS response time or age.^31^.

Prior to the COVID-19 pandemic, previous studies have examined suicide-related OHCAs. In comparison to our suicide-related OHCA characteristics, investigators from Korea reported a higher overall incidence rate, higher rates in older individuals, more unwitnessed cases, higher numbers of asystolic cases, and a higher survival rate.5., 8. They demonstrated similarities with our study with a predominance of male cases and high rates of hanging. Australia data examining OHCAs with hanging (2000–2017), showed increases in overall incidence and bystander CPR, and a decrease in witnessed arrests over time.^6^ Similar to our study, there were no significant differences in ROSC, non-shockable rhythm, or survival rate. Only 23% of hanging-related OHCA were EMS-treated, which may partially account for the relatively high EMS-treated survival rate (2.9%).

Our observational study has limitations. Our findings may not be generalizable to other areas due to varying COVID-19 public health orders, vaccine distribution, incidence rate of COVID-19, available mental health resources, government pandemic response, and patient characteristics. It is possible that COVID-related changes in EMS protocols (Appendix B) may have influenced suicide-related OHCA outcomes. It is possible that incomplete clinical information may have been available to paramedics and thus we may have underestimated the true suicide-related OHCA rate (for example, an overdose of an illicit substance with no other historical details may have actually been intentional, but would not be classified as such as this information was not known). We lacked data on blunt or penetrating-related suicides. However, the primary goal of this study was not to determine the true community-level incidence of suicide, but rather to identify changes in incidence before and during the COVID pandemic, using a standardized definition, in order to estimate whether suicide may be playing a role in COVID-related increases seen in non-traumatic OHCA registries. In addition, cases of homicide may have been mis-classified as suicide.

## Conclusion

In British Columbia, the incidence of suicide-related non-traumatic OHCA declined by one per 100 000 person-years during the COVID-19 pandemic and we did not detect changes in outcomes. These data suggesting that suicide is not playing a role in increases in COVID-related OHCA incidence or mortality. Overall survival rates of suicide-related OHCA are very low.

## Funding

This analysis was supported by Biotalent Canada.

## Declaration of Competing Interest

The authors declare that they have no known competing financial interests or personal relationships that could have appeared to influence the work reported in this paper.
