# Supplementary material for: Temporal trends of suicide-related non-traumatic out-of-hospital cardiac arrest characteristics and outcomes with the COVID-19 pandemic
Source: Resusc Plus. 2022 Mar 3;9:100216. doi: 10.1016/j.resplu.2022.100216 (PMC8890978; doi:10.1016/j.resplu.2022.100216)
Supplement: Supplementary data 1 [file mmc1.pdf]

Appendix A

BC Emergency Health Services Policy on Withholding Resuscitative Treatments[1]

Policy dictates that all patients must be provided resuscitative treatments for cardiac arrest except in the following circumstances:

“(1) “Obvious Death” defined as rigor mortis, decapitation, Hypostasis (lividity), tissue decomposition, thoracic or abdominal transection, incineration of the torso or head, or complete destruction or removal of vital organs

(2) If performing CPR endangers the life, safety, or health of paramedics/fire department

(3) There is a “No CPR” order in effect; for example, advance directive or medical order for scope of treatment (MOST)

Appendix B

BC Emergency Health Services COVID-19 policy updates for OHCAs[2]

(1) CPR changes

   a. Paramedics must wear airborne Personal Protective Equipment (N95/EHFR, face shield, gown, gloves)
   b. Surgical masks placed over patient's face before CPR performed
   c. Emergency medical dispatch advising bystander chest compressions-only CPR and covering patients mouth and face

(2) Ventilation changes

   a. Supraglottic airway (iGel) with viral filter should be used as the main device for airway management
   b. Intubations are reserved for only when paramedics are unable to maintain a proper seal with supraglottic airway and other means of oxygenating the patient are ineffective.

(3) Discontinuation changes

   a. Contact Clinical Support at 15 mins or less if early discontinuation criteria fulfilled:
      i. Unwitnessed OHCA
      ii. No shocks
      iii. No ROSC

References:

1. R01: Commencing and Withholding Resuscitation. https://handbook.bcehs.ca/clinical-practice-guidelines/r-resuscitation-decision-making/r01-commencing-and-withholding-resuscitation/. Accessed October 6, 2021.
2. COVID-19 Summary of Clinical Practice Changes. https://handbook.bcehs.ca/clinical-resources/covid-19/covid-19-summary-of-clinical-practice-changes/. Accessed September 28, 2021.
